# Hepatic Artery Infusion Chemotherapy for Hepatocellular Carcinoma: Clinical Advancements

**DOI:** 10.3390/curroncol32060313

**Published:** 2025-05-28

**Authors:** Wei Xu, Qing Li, Bin Liang

**Affiliations:** 1 Hubei Provincial Clinical Research Center for Precision Radiology & Interventional Medicine, Hubei Key Laboratory of Molecular Imaging, Department of Radiology, Union Hospital, Tongji Medical College, Huazhong University of Science and Technology, 1277 Jiefang Road, Wuhan 430022, China; m202476500@hust.edu.cn; 2 Department of Radiology, The First Affiliated Hospital of Soochow University, Suzhou 215006, China; 20224032007@stu.sa.edu.cn

**Keywords:** hepatocellular carcinoma, hepatic artery infusion chemotherapy, locoregional therapy, combination therapy

## Abstract

Intermediate- and advanced-stage hepatocellular carcinoma (HCC) continues to present significant therapeutic challenges. Hepatic artery infusion chemotherapy (HAIC), a well-established locoregional treatment for unresectable HCC, has recently demonstrated promising clinical outcomes both as monotherapy and in combination with systemic therapies. This comprehensive review examines recent clinical advances in HAIC for HCC, with particular emphasis on evolving treatment regimens and their therapeutic efficacy.

## 1. Introduction

Hepatocellular carcinoma (HCC) stands as a major global health burden, ranking among the most prevalent malignancies and representing the third leading cause of cancer-related mortality worldwide [1]. China bears a disproportionate share of this disease burden, contributing nearly 50% of global HCC incidence [2]. Alarmingly, the majority of patients present with intermediate- or advanced-stage disease at diagnosis, rendering them ineligible for potentially curative interventions including surgical resection, liver transplantation, or ablation [2]. Current therapeutic paradigms guided by the Barcelona Clinic Liver Cancer (BCLC) staging system recommend transarterial chemoembolization (TACE) for intermediate-stage disease and identify Atezolizumab–Bevacizumab or Durvalumab–Tremelimumab as first-line systemic therapies for advanced cases [3]. However, suboptimal response rates and frequent disease progression underscore the critical need for innovative treatment strategies combining locoregional and systemic approaches [4,5].

Hepatic artery infusion chemotherapy (HAIC), also known as transcatheter arterial infusion (TAI), has re-emerged as a promising locoregional modality, driven by advancements in transcatheter techniques and optimized chemotherapeutic regimens. Modern HAIC protocols demonstrate tumor response rates exceeding 50% in selected populations, with particular efficacy in cases featuring portal vein invasion [6,7]. The therapeutic landscape has been further transformed by emerging evidence supporting synergistic effects when combining HAIC with molecular targeted agents (e.g., sorafenib, lenvatinib) and immune checkpoint inhibitors, achieving remarkable median overall survival (OS) durations exceeding 17 months in advanced HCC cohorts [6,7]. This review critically examines contemporary clinical advances in HAIC application for HCC, with particular emphasis on evolving treatment protocols, combination strategies, and their associated therapeutic outcomes. To ensure clinical relevance, this review synthesizes key evidence published from 1999 to 2024, encompassing clinical trials, consensus guidelines, and technical studies, with particular emphasis on Phase III trials and large cohort studies that have informed protocol optimization and regional practice patterns.

## 2. Definition and Technical Modalities

### 2.1. Definition

HAIC involves the direct administration of chemotherapeutic agents into the hepatic arterial system via an intra-arterial catheter. This targeted delivery mechanism achieves higher intratumoral drug concentrations compared to systemic chemotherapy while reducing extrahepatic toxicity [8]. As a specialized regional chemotherapy approach, HAIC fundamentally differs from conventional intravenous chemotherapy through its first-pass hepatic extraction advantage.

### 2.2. Technical Modalities

#### 2.2.1. Surgical Pump Implantation

The surgically implanted subcutaneous pump remains the gold-standard technique in Western practice. This procedure requires laparotomy or laparoscopy under direct visualization to ensure precise catheter placement. Specifically, the catheter is inserted retrograde into the gastroduodenal artery (GDA) and secured with sutures. During the procedure, extrahepatic arterial branches are ligated to prevent extrahepatic chemotherapeutic exposure, while accessory hepatic arteries are occluded to minimize competitive intrahepatic flow. The catheter tip is precisely positioned at the GDA–common hepatic artery (CHA) junction to optimize hepatic perfusion. Methylene blue is commonly used to verify homogeneous hepatic perfusion and exclude inadvertent extrahepatic perfusion. Finally, the pump is connected to the catheter and implanted in a subcutaneous pocket [9,10]. In most centers, post-implantation nuclear medicine studies are routinely conducted before initiating HAIC to ensure safety.

While enabling repeated treatments, this technique carries significant limitations. First, the procedure requires laparotomy or laparoscopy performed by experienced surgeons and typically requires concomitant cholecystectomy, resulting in substantial invasiveness and potential surgical complications [11]. Second, although preoperative CT can assess hepatic arterial anatomy and aberrant vessels, this technique demonstrates limited control of collateral tumor supply (e.g., subphrenic artery). Thirdly, catheter placement into arteries beyond the GDA is occasionally required, posing considerable technical difficulties [12].

#### 2.2.2. Transfemoral Temporary Catheterization

The transfemoral temporary catheterization has been widely adopted in Chinese clinical practice [13]. This percutaneous approach involves the percutaneous insertion of a catheter in the proper hepatic artery or the tumor-feeding hepatic artery branch under digital subtraction angiography (DSA) guidance. The external end of the catheter is retained outside the skin of the arterial approach and connected with an infusion pump for chemotherapy. After the completion of a single session of chemotherapy, the catheter was removed with the puncture arterial hemostasis. It is noted that before the indwelling catheter placement, routine arterial angiography is performed to evaluate hepatic arterial anatomy and tumor blood supply. When necessary, vascular embolization is employed to address hepatic artery variations or extrahepatic collateral blood supply, thereby ensuring selective drug distribution to the liver or the tumor [14]. This procedure needs to be repeated according to the HAIC treatment schedule.

As a minimally invasive interventional procedure, this technique offers several advantages, including procedural simplicity and rapid postoperative recovery. The flexibility to adjust catheter positioning according to therapeutic requirements further enhances its clinical utility. However, certain limitations should be noted. Firstly, repeated catheterization and bedridden infusion chemotherapy may impair patient tolerance and compliance. Secondly, the tip of the indwelling catheter may become dislocated due to vomiting, coughing, or drastic positional changes, resulting in improper drug delivery. Finally, due to the limited duration of catheter placement, this approach is unsuitable for chemotherapy regimens requiring prolonged infusion or repeated short-term administration [11].

#### 2.2.3. Percutaneous Port Systems

The percutaneous port systems represent a significant advancement in HAIC technology, with widespread clinical adoption across Asian regions. This minimally invasive procedure employs the “tip-fixation” technique to precisely position an indwelling side-holed catheter within the GDA. The catheter is strategically placed with its side-hole aligned at the origin of the common hepatic artery. Through a coaxial approach, a microcatheter is advanced via the indwelling catheter, passing through the side-hole for optimal positioning. The distal tip of the indwelling catheter is then securely anchored within the GDA using either embolization coils or an NBCA–lipiodol mixture. Finally, the proximal end of the indwelling catheter is connected to an implantable port system for drug infusion [15,16,17].

**Figure 1:**
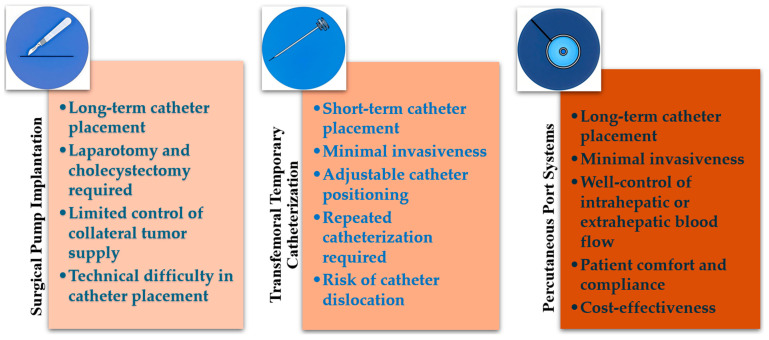
Features of the techniques for HAIC.

This technique integrates the advantages of previous HAIC techniques, facilitating routine angiography and necessary procedures to redistribute intrahepatic or extrahepatic blood flow, thereby enhancing the efficacy and safety of chemotherapy. Additionally, it allows for multiple treatments with a single placement, accommodating various chemotherapy regimens, improving patient comfort and compliance, and reducing overall treatment costs [18,19,20,21,22]. The main features of the above three techniques are summarized in Figure 1.

## 3. Pharmacological Rationale and Chemotherapeutic Agents

### 3.1. Pharmacological Rationale

The therapeutic foundation of HAIC stems from the differential vascular perfusion between hepatocellular malignancies and parenchymal tissue. While normal hepatocytes receive approximately 75% of their blood supply through the portal venous system, hepatic malignancies derive >90% of their perfusion from arterial circulation [23]. Therefore, it is reasonable to use the hepatic artery as an approach to deliver concentrated doses of chemotherapy to the tumor bed.

Mechanistically, HAIC capitalizes on two key pharmacokinetic advantages: first-pass hepatic extraction and enhanced tumor penetration. Studies have demonstrated that hepatic arterial infusion of floxuridine or 5-fluorouracil can achieve intrahepatic uptake rates of up to 90% and 19–90%, respectively. These rates significantly exceed those observed with conventional intravenous administration. Accordingly, intratumoral drug concentrations are also markedly elevated [9,24]. In addition, sustained high-flow infusion creates increased interstitial pressure gradients, improving intratumoral drug distribution [25]. These synergistic mechanisms augment the efficacy of chemotherapy while mitigating extrahepatic toxicity.

### 3.2. Drug Selection

HAIC drug selection requires strategic integration of systemic chemotherapy principles with arterial pharmacokinetic advantages. Priority should be given to tumor-sensitive drugs and prototype drugs, with the use of combinations or sequential regimens of agents with differing mechanisms strategically designed to optimize the therapeutic efficacy [26,27]. Drugs that share similar toxic effects or exhibit cumulative hepatotoxicity, as well as those with antagonistic pharmacological effects or the potential to inactivate one another, should be avoided. The primary objective is to minimize toxicity while maximizing therapeutic efficacy against the tumor and decreasing side effects both systemically and within the liver.

Cell cycle-nonspecific agents (e.g., alkylating agents, anthracyclines, and platinum complexes) exhibit concentration-dependent cytotoxicity. These agents mandate high-intensity bolus administration protocols. For instance, oxaliplatin is usually administered short-term, high-dose infusion (85–130 mg/m^2^ over 90–120 min). In contrast, cell cycle-specific agents (e.g., 5-fluorouracil and floxuridine) are time-dependent, requiring sustained tumor exposure to achieve maximal cytotoxic effects. These agents necessitate precision-controlled infusion protocols. For instance, 5-fluorouracil is usually administered with continuous infusion (2400 mg/m^2^ over 46 h) [28,29].

## 4. Patient Selection and Preprocedural Evaluation

### 4.1. Patient Selection

HAIC is primarily indicated for patients with HCC presenting with multifocal intrahepatic lesions or bulky tumors, portal vein thrombosis, Child-Pugh class A/B liver function, and an Eastern Cooperative Oncology Group (ECOG) performance status of 0–2. It may also serve as an alternative therapeutic option for advanced-stage patients with limited extrahepatic metastases [6,7].

Current clinical guidelines endorse HAIC for HCC management. The Japanese Society of Hepatology (JSH) guidelines recommend HAIC for patients with ≥4 intrahepatic lesions in the absence of vascular invasion [5]. Similarly, the Korean Liver Cancer Association–National Cancer Center (KLCA-NCC) guidelines propose HAIC for patients with portal vein invasion and no extrahepatic spread who have failed or are ineligible for first- or second-line systemic therapies [30]. Notably, the Chinese Society of Clinical Oncology (CSCO) guidelines have expanded HAIC indications to include (1) Stage Ib–IIb patients with solitary tumors >7 cm who are ineligible for or decline surgical resection; (2) Stage III patients refractory to or declining molecular targeted therapy/systemic chemotherapy; (3) Stage IIIb patients with limited extrahepatic metastases (treatment decision at clinician’s discretion); and (4) Stage IV patients unwilling or unable to undergo liver transplantation [13].

However, HAIC remains predominantly utilized in Asian countries and is excluded from major international guidelines, primarily due to concerns that HAIC may exacerbate liver dysfunction, particularly in patients with underlying cirrhosis [31]. Severe liver function impairment from treatment could reduce life expectancy, while even mild hepatic deterioration might compromise subsequent therapies. The absence of reliable predictive biomarkers or scoring systems for HAIC response necessitates careful evaluation of liver function reserve during patient selection [31].

### 4.2. Preprocedural Evaluation

Before the initiation of HAIC, a comprehensive patient evaluation should be conducted, encompassing detailed medical history and physical examination, laboratory analyses (hepatic/renal function, coagulation profile, complete blood count, tumor markers), and contrast-enhanced dynamic CT or MR imaging. Positron emission tomography–computed tomography (PET-CT) may supplement diagnostic workup when indicated. Liver biopsy is recommended for histopathological confirmation in cases with an inconclusive HCC diagnosis or suspected alternative hepatic malignancies.

Key preoperative parameters include liver function, performance status, and tumor burden. Regarding liver function, clinical trials predominantly enroll patients with compensated cirrhosis (Child-Pugh A to B7) for HAIC monotherapy or combination regimens [32,33]. Real-world evidence demonstrates acceptable HAIC tolerability in Child-Pugh B patients [6]. Similarly, most trials restrict HAIC eligibility to Eastern Cooperative Oncology Group (ECOG) 0–1 [7], while observational studies extend criteria to ECOG 0–2 [6]. In terms of tumor burden, HAIC is indicated for multifocal intrahepatic lesions or bulky tumors with portal vein thrombosis. Notably, therapeutic efficacy diminishes significantly when tumor involvement exceeds ≥50% of total liver volume [34].

## 5. HAIC Chemotherapy Regimens and Outcomes

Current clinical HAIC protocols include FOLFOX, low-dose fluorouracil–cisplatin (FP), fluorouracil–interferon arterial infusion therapy (FAIT), New FP, and oxaliplatin–raltitrexed regimens, with efficacy outcomes varying by drug combination. The included studies of HAIC combination therapies and their characteristics are summarized in Table 1. The efficiency and safety in the major studies of HAIC monotherapies are summarized in Table 2.

### 5.1. FOLFOX Regimen

**Figure 2:**
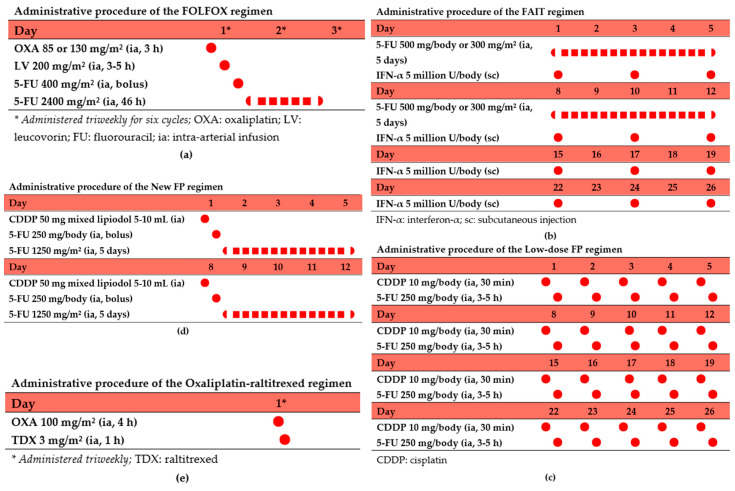
Administrative procedures of the main regimens for HAIC: (**a**) FOLFOX, (**b**) FAIT, (**c**) low-dose FP, (**d**) new FP, and (**e**) oxaliplatin–raltitrexed.

The FOLFOX regimen is the recommended first-line systemic chemotherapy for HCC in China and has been widely adopted in HAIC treatment [13]. The standard FOLFOX regimen for HAIC includes oxaliplatin (85–130 mg/m^2^ via 3 h intra-arterial infusion on day 1), leucovorin (200 mg/m^2^ via 3–5 h intra-arterial infusion on day 1), and fluorouracil (400 mg/m^2^ intra-arterial bolus, followed by 2400 mg/m^2^ 46 h continuous infusion). Treatment is typically administered every three weeks for six cycles, with adjustments made based on tumor response (Figure 2a) [7]. The FOLFOX regimen synergizes platinum concentration-dependent effects with fluorouracil time-dependent cytotoxicity, enhanced by leucovorin-mediated biochemical modulation.

HAIC monotherapy has demonstrated significant survival benefits for patients with advanced HCC. A prospective, non-randomized Phase II study compared the efficacy of HAIC and TACE in patients with massive unresectable HCC. The results showed that HAIC achieved significantly higher partial response rates and disease control rates compared to TACE (52.6% vs. 9.8%, *p* < 0.001; 83.8% vs. 52.5%, *p* < 0.01) [47]. Another randomized controlled Phase III trial evaluated HAIC versus TACE in patients with unresectable HCC without vascular invasion or extrahepatic metastasis. HAIC as a first-line treatment significantly improved OS (23.1 vs. 16.1 months, *p* < 0.001) and reduced the incidence of severe adverse events (AEs) (19% vs. 30%, *p* = 0.03) [48]. A recent randomized controlled Phase III trial comparing HAIC with sorafenib as first-line therapy for advanced HCC revealed that HAIC significantly prolonged OS compared to sorafenib (13.9 vs. 8.2 months, *p* < 0.001) [32]. Additionally, a Phase III, multicenter, prospective, open-label, randomized controlled trial compared postoperative adjuvant HAIC with routine follow-up in patients with HCC with microvascular invasion. HAIC significantly extended median disease-free survival (20.3 vs. 10.0 months, *p* < 0.001) [49].

HAIC combined with targeted therapy has also emerged as a viable option for advanced HCC. An earlier randomized controlled trial compared sorafenib plus HAIC to sorafenib alone in patients with advanced HCC and portal vein invasion. The combination therapy resulted in longer OS (13.37 vs. 7.13 months, *p* < 0.001), longer progression-free survival (PFS) (7.03 vs. 2.6 months, *p* < 0.001), and a higher tumor response rate (40.8% vs. 2.46%, *p* < 0.001), although grade 3/4 AEs were more frequent in the combination group [35]. Another Phase II clinical trial confirmed the superior OS of sorafenib plus HAIC compared to sorafenib alone (16.3 vs. 6.5 months, *p* < 0.001) in advanced HCC with major portal vein tumor thrombosis [33].

HAIC combined with immunotherapy, as well as with both targeted therapy and immunotherapy, has also shown promising efficacy and safety [36,37,38,39]. A Phase II, single-center, single-arm study treated advanced, high-risk HCC patients with a combination of lenvatinib, toripalimab, and HAIC, achieving a 6-month PFS rate of 80.6% [38]. Another single-arm Phase II clinical study evaluated HAIC combined with camrelizumab and apatinib for advanced HCC, reporting an objective response rate of 77.1% and a median PFS of 10.38 months [39].

Other combination therapies, such as HAIC plus TACE or ablation, have also been explored [40,41]. A Phase II, prospective, non-randomized clinical study compared TACE combined with HAIC to TACE alone in patients with unresectable HCC without extrahepatic metastasis. The combination therapy showed significant improvements in overall response rate and median PFS (68.9% vs. 45.9%, *p* < 0.05; 8 vs. 4.5 months, *p* < 0.001) [41]. Additional clinical trials of combination therapies are currently underway.

### 5.2. Low-Dose FP Regimen

The FP regimen, pioneered by Japanese researchers for HAIC, employs daily low-dose cisplatin (10 mg/d via 30 min intra-arterial infusion) followed by fluorouracil (250 mg/d over 3–5 h). Administered 5 consecutive days/week with 2-day intervals per 4-week cycle (Figure 2b), this protocol utilizes cisplatin as a biochemical modulator to enhance fluorouracil’s antitumor activity through intracellular reduced folate accumulation, achieving synergistic cytotoxicity. Its brief infusion requirements facilitate compatibility with percutaneous port–catheter systems [64].

As a monotherapy, the FP-HAIC regimen demonstrated 20–71% tumor response rates and 7.3- to 15.9-month median OS in advanced HCC with portal vein invasion in early retrospective analyses [50,51,52,53,64,65,66,67]. The combination of FP-HAIC with targeted therapy has also been investigated. In a Phase I/II trial, the FP-HAIC combined with sorafenib demonstrated a response rate of 38.9%, a disease control rate of 77.8%, a median time-to-progression of 9.7 months, and a 1-year survival rate of 88.2% [42]. Another open-label, non-comparative Phase II trial using HAIC or HAIC followed by sorafenib for patients with advanced HCC revealed 1-year and 2-year survival rates of 64.0% and 48.3%, respectively [43]. A recent randomized controlled Phase III trial compared the FP-HAIC combined with sorafenib to sorafenib monotherapy in advanced HCC. While no statistically significant difference in OS was observed between the two groups (11.8 vs. 11.5 months, *p* = 0.955), subgroup analysis indicated that patients with main portal vein invasion who received the combination therapy had significantly longer median OS compared to those receiving sorafenib alone (11.4 vs. 6.5 months, *p* = 0.05) [44]. These data position FP-HAIC combination therapy as a potential survival-enhancing strategy for portal vein-involved HCC.

### 5.3. FAIT Regimen

The FAIT regimen was initially described in 2002 for HCC management [68]. This regimen combines intra-arterial infusion of fluorouracil with subcutaneous injection of interferon. This therapeutic protocol involves transarterial administration of fluorouracil coupled with subcutaneous interferon-α injections. Fluorouracil is delivered through continuous intra-arterial infusion according to two distinct dosing schemes: 500 mg/day or 300 mg/m^2^/day, administered five consecutive days weekly during the first two weeks, followed by a two-week break. Subcutaneous interferon-α is administered at 5 million IU/day, three times weekly for four weeks, with a total of 1–4 treatment cycles (Figure 2c).

Clinical investigations have documented objective response rates (ORRs) of 24.6–73.0% and median OS durations of 6.9–14.7 months in patients with HCC with portal vein tumor thrombosis (PVTT) treated with FAIT [54,55,56,57,69,70,71,72,73]. A Phase II trial revealed significant enhancement of therapeutic outcomes through cisplatin–FAIT combination therapy, demonstrating superior ORR (45.6% vs. 24.6%; *p* = 0.03) and extended median OS (17.6 vs. 10.5 months) compared with FAIT monotherapy [71].

### 5.4. New FP Regimen

Initially, cisplatin was administered as monotherapy via hepatic artery at a dose of 65 mg/m^2^ for advanced HCC, but its clinical efficacy was limited [74,75]. Subsequent studies combined this regimen with sorafenib, demonstrating superior outcomes compared to sorafenib monotherapy [76,77]. Further exploration of cisplatin-based combination therapies led to the Phase II LEOPARD trial, which evaluated lenvatinib plus HAIC with cisplatin. This combination achieved an ORR of 64.7% (95% CI: 46.5–80.3%) by mRECIST and 45.7% (95% CI: 28.8–63.4%) by RECIST 1.1 [78].

The new FP regimen combines cisplatin, lipiodol, and fluorouracil. The protocol involves intra-arterial administration of 50 mg cisplatin emulsified with 5–10 mL lipiodol, followed by a bolus injection of 250 mg fluorouracil and a continuous infusion of 1250 mg/m^2^ fluorouracil over five days, with a two-day rest period (Figure 2d). Treatment is administered weekly for two or three consecutive weeks. In this regimen, the therapeutic efficacy of cisplatin is enhanced by the tumor-targeting properties of lipiodol, while the dose of fluorouracil is increased to maximize antitumor effects.

Retrospective analyses have demonstrated superior median OS in patients with advanced HCC with macrovascular invasion treated with the new FP regimen compared to low-dose FP or sorafenib (24.7 vs. 16.1 months; *p* < 0.05; and 18.0 vs. 9.0 months; *p* < 0.0001, respectively) [58,59]. A multicenter, single-arm Phase II trial evaluating this regimen in HCC with PVTT reported a median disease-free survival of 8.6 months, OS of 27.0 months, and ORR of 75% [60]. Synergistic efficacy was observed when combined with lenvatinib, achieving an ORR of 83% in advanced HCC [45].

A non-randomized prospective cohort study comparing the new FP regimen to sorafenib in HCC with macrovascular invasion revealed significant improvements in OS (30.4 vs. 13.2 months; *p* = 0.013) and ORR (71% vs. 10%; *p* < 0.001) [79]. These outcomes are corroborated by recent large-scale multicenter retrospective studies, wherein HAIC using the new FP regimen extended median OS in locally advanced HCC versus sorafenib (12.0 vs. 7.9 months; *p* < 0.001) [61].

### 5.5. Oxaliplatin–Raltitrexed Regimen

The clinical utility of fluorouracil is constrained by its short plasma half-life, necessitating prolonged continuous infusions or repeated short-term administrations to exploit its time-dependent cytotoxic activity. These delivery modalities, however, impose significant logistical challenges for patients undergoing HAIC. Raltitrexed, a novel thymidylate synthase inhibitor, has been investigated as a potential HAIC agent for HCC. Its extended plasma half-life relative to fluorouracil may improve treatment tolerability when combined with platinum-based chemotherapeutics [62].

A single-arm Phase II trial assessing oxaliplatin–raltitrexed HAIC in intermediate-to-advanced HCC documented an ORR of 51.4%, with median PFS of 6.7 months, median disease-free survival (DFS) of 5.2 months, and 1-year survival rate of 43.2%. The protocol comprised oxaliplatin (100 mg/m^2^) delivered via 4 h intra-arterial infusion and raltitrexed (3 mg/m^2^) administered over 60 min, significantly reducing the total infusion duration. Treatment cycles were repeated triweekly (Figure 2e), with no grade ≥4 treatment-related adverse events (TRAEs) reported [63]. Another Phase II study evaluating apatinib combined with oxaliplatin–raltitrexed HAIC in patients with HCC with extrahepatic metastases refractory to first-line systemic therapy achieved an ORR of 53.8% [46].

## 6. Adverse Events and Their Management

AEs associated with HAIC are systematically classified into chemotherapy-induced toxicities and procedural complications [9,80,81].

### 6.1. Chemotherapy-Induced AEs

Gastrointestinal disturbances, predominantly nausea and vomiting (incidence >30%), represent the most frequent chemotherapy-related AEs [9]. These manifestations are typically managed effectively through prophylactic antiemetic regimens. Acute epigastric pain, frequently attributable to chemotherapy-induced arterial vasospasm, may necessitate transient infusion cessation or intra-arterial lidocaine administration for symptomatic relief. Hematological toxicity, manifesting as leukopenia and thrombocytopenia, often requires granulocyte colony-stimulating factor therapy, thrombopoietin receptor agonists, or partial splenic embolization in refractory cases. Hepatotoxicity, evidenced by elevated serum alanine aminotransferase and total bilirubin, mandates hepatoprotective agents such as ursodeoxycholic acid or polyene phosphatidylcholine. It is worth mentioning that transient post-procedural aminotransferase elevation, reflecting tumor necrosis without hepatic functional compromise, may predict cTACE response [82]. Whether elevated serum aminotransferases can serve as a prognostic marker associated with treatment response requires further validation. Nephrotoxicity prophylaxis is achieved through standardized hydration protocols during cytotoxic drug infusion. Cardiotoxicity and gastroduodenal mucosal injury, though less common, warrant systematic monitoring with adjunctive interventions including myocardial metabolic support and proton pump inhibitor therapy.

### 6.2. HAIC Procedure-Related AEs

HAIC procedure-associated complications are principally stratified into implantable pump-related morbidities and catheter-associated events [80,81]. Implantable pump complications (incidence: 8–18%) predominantly comprise pocket hematoma, device-related infections, pump erosion, mechanical displacement (migration/flipping), and reservoir exposure [81]. Minor hematomas are typically managed nonoperatively through compression bandages and pressure garments. Infectious complications (abscess formation or cellulitis) generally require ultrasound-guided drainage coupled with empirical antibiotic therapy; recalcitrant cases may necessitate pump explantation with delayed reimplantation [83]. Pump displacement is corrected via surgical repositioning, whereas full-thickness erosion with cutaneous penetration mandates complete device removal and alternate-site implantation. Catheter-related complications (10–26% incidence) primarily involve thrombosis (occlusion), mechanical dislodgement, or mural erosion [81,84]. These complications can usually be resolved through thrombolysis, catheter repositioning or replacement, or embolization treatment.

## 7. Conclusions

HAIC has demonstrated preliminary efficacy as a safe and technically feasible therapeutic modality for HCC. While widely adopted in China and select Asian nations, HAIC remains excluded from global HCC treatment guidelines. To establish HAIC as a globally recognized HCC treatment paradigm, three strategic imperatives may need to be prioritized: (1) development of consensus-driven technical guidelines through international multicenter clinical trials and Delphi-method expert panels; (2) optimization of tumor biology-informed HAIC regimens using pharmacogenomic profiling and radiomic response predictors; and (3) mechanistic exploration of HAIC combined with immunotherapy/molecular targeted therapy through preclinical models correlating intratumoral drug distribution with immune microenvironment modulation. Continued innovation in HAIC may advance precision HCC management, ultimately improving oncological outcomes.

## Figures and Tables

**Table 1:** Summary of included studies of HAIC combination therapies.

Study Design	Patient Population	Arm	Sample Size	Efficacy Outcomes	Author (Years)	References
RCT Phase II	Advanced HCC with major PVTT	HAIC (FOLFOX) + Sorafenib	32	OS: 16.3 months; ORR: 41%, PFS: 9.0 months	Zeng, K. (2022)	[33]
		Sorafenib	32	OS: 6.5 months; ORR: 3%, PFS: 2.5 months		
RCT Phase III	HCC with PVI (Vp3 and Vp4)	HAIC (FOLFOX) + Sorafenib	125	OS: 13.37 months; ORR: 40.8%, PFS: 7.03 months	He, M. (2019)	[35]
		Sorafenib	122	OS: 7.13 months; ORR: 2.46%, PFS: 2.6 months		
Retrospective Analysis	Intermediate and advanced HCC unsuitable for TACE	HAIC (FOLFOX) + PD-(L)1 Inhibitors + MTT	55	OS: 15.0 months, PFS: 10.0 months, ORR: 43.6%, DCR: 61.8%	Tang, H.-H. (2023)	[36]
Retrospective Analysis	Unresectable HCC suitable for HAIC or TACE	HAIC (FOLFOX) + TKIs + PD-(L)1 Inhibitors	302	OS: Not reached, PFS: 12.4 months, ORR: 33.1%, DCR: 77.8%	Yu, B. (2023)	[37]
		TACE + TKIs + PD-(L)1 Inhibitors	446	OS: 13.8 months, PFS: 8.2 months, ORR: 7.8%; DCR: 47.1%		
Single-arm Phase II	Advanced HCC unsuitable for TACE	HAIC (FOLFOX) + Lenvatinib + Toripalimab	36	PFS at 6 months: 80.6%, median PFS: 10.4 months, median OS: 17.9 months	Lai, Z. (2022)	[38]
Single-arm Phase II	Intermediate and advanced HCC unsuitable for TACE	HAIC-FOLFOX + Camrelizumab + Apatinib	35	ORR: 77.1%, DCR: 97.1%, median PFS: 10.38 months	Zhang, T.-Q. (2023)	[39]
Retrospective Analysis	Large HCC	HAIC (FOLFOX)	135	OS: 14.5 months, PFS: 4.6 months, ORR: 33.1%	You, H. (2022)	[40]
		HAIC (FOLFOX) and sequential ablation	93	OS: 22.2 months, PFS: 8.5 months, ORR: 51.4%		
RCT Phase II	Inoperable HCC without distant metastasis	Chemoembolization alone	39	ORR: 45.9%, mPFS: 4.5 months	Gao, S. (2015)	[41]
		HAIC (FOLFOX) + Chemoembolization	45	ORR: 68.9%, mPFS: 8.0 months		
Single-arm Phase I/II	Advanced HCC	HAIC (Low-dose FP) + Sorafenib	18	ORR: 38.9%, DCR: 77.8%, TTP: 9.7 months, 1-year OS: 88.2%	Ueshima, K. (2015)	[42]
Single-arm Phase II	Advanced HCC	HAIC (Low-dose FP) followed by sorafenib if non-response	55	1-year OS: 64.0%, 2-year OS: 48.3%	Hatooka, M. (2018)	[43]
RCT Phase III	Advanced HCC	Sorafenib	103	OS: 11.5 months	Kudo, M. (2018)	[44]
		HAIC (Low-dose FP) + Sorafenib	103	OS: 11.8 months		
Retrospective Analysis	Unresectable HCC with prior systemic therapy	HAIC (New FP) + Lenvatinib	6	ORR: 83%, DCR: 100%	Maruta, S. (2024)	[45]
Single-arm Phase II	Advanced HCC with extrahepatic metastasis	HAIC (Oxaliplatin–raltitrexed) + Apatinib	39	ORR: 53.8%; PFS: 6.2 months, OS: 11.3 months, DCR: 89.7%	Chen, S. (2024)	[46]

RCT: randomized controlled trial; PVTT: portal vein tumor thrombosis; PVI: portal vein invasion; PD-(L)1 inhibitors: programmed death-1 or programmed death-ligand 1 inhibitors; MTT: molecular targeted therapy; TKIs: tyrosine kinase inhibitors; OS: overall survival; ORR: objective response rate; PFS: progression-free survival; DCR: disease control rate; mPFS: median progression-free survival; TTP: time to progression.

**Table 2:** Efficiency and safety of major studies of HAIC monotherapy.

Regimen	Study Design	Sample Size	Patient Population	OS (mo)	PFS (mo)	ORR (%)	DCR (%)	Severe * or Gr 3–4 AEs (%)	Major Gr 3–4 AEs (%)	Author (Years)	References
**FOLFOX**	RCT Phase III	130	Advanced HCC	13.9	7.8	31.5	**-**	20.3	Elevated AST (10.9); thrombocytopenia (10.9)	Lyu, N. (2022)	[32]
	Prospective Phase II	38	Unresectable HCC	**-**	TTP: 5.87	54.1	83.8	34.2	Vomiting (10.5); leukopenia (7.9)	He, M. (2017)	[47]
	RCT Phase III	159	Unresectable HCC	23.1	9.6	46 .0	82	19.0 *	Elevated AST (17.8); elevated ALT (8.3)	Li, Q.-J. (2022)	[48]
	RCT Phase III	157	HCC with MVI post-op	3-year OS rate: 80.4%	DFS: 20.3	**-**	**-**	**-**	Pain (1.4)	Li, S.-H. (2023)	[49]
**Low-dose FP**	Retrospective	32	Advanced HCC	10.3	TTF: 3.6	31.3	56.3	-	Thrombocytopenia (25.0); neutropenia (12.5)	Moriguchi, M. (2017)	[50]
	Retrospective	48	HCC with PVTT	3-year OS rate: 25%	-	48	77	-	-	Ando, E. (2002)	[51]
	Retrospective	71	Advanced HCC	10.2	-	35	-	-	Leukocytopenia (13.0)	Niizeki, T. (2012)	[52]
	Nationwide Survey	476	Advanced HCC	14	-	40.5	-	-	-	Nouso, K. (2013)	[53]
**FAIT**	Prospective Phase II	59	HCC with PVTT	29.9	9.7	73	91.6	-	Leucopenia (10.1); thrombocytopenia (8.4)	Kasai, K. (2012)	[54]
	Prospective Phase II	55	HCC with major PVTT	11.8	-	43.6	50.9	14.6	Thrombocytopenia (9.1); leukopenia (5.5)	Ota, H. (2005)	[55]
	Retrospective	116	HCC with PVI	6.9	-	52.6	54.3	-	-	Obi, S. (2006)	[56]
	RCT Phase II	30	Advanced HCC	8.4	3.5	26.7	63.3	51.6 *	Leukopenia (32.3); thrombocytopenia (29.0); neutropenia (29.0)	Monden, M. (2012)	[57]
**New FP**	Retrospective	99	HCC with MVI without EHS	24.7	8.8	76	88	26.2 *	Thrombocytopenia (8.1); cholangitis (6.1)	Niizeki, T. (2021)	[58]
	Retrospective	671	HCC	18	-	73	-	-	-	Iwamoto, H. (2022)	[59]
	Prospective Phase II	52	HCC with PVTT	27	8.6	75	96.2	-	Renal dysfunction (4.0); CDDP allergy (4.0)	Nagamatsu, H. (2016)	[60]
	Retrospective	644	HCC	12	-	-	-	-	-	Iwamoto, H. (2021)	[61]
**Oxaliplatin–raltitrexed**	RCT Phase II	61	Unresectable CRCLM	13.1	4.6	22.4	71.4	-	Abdominal pain (50.8)	Feng, A.-W. (2022)	[62]
	Prospective Phase II	39	Intermediate and advanced HCC	1-year OS rate: 43.2%	5.2	46.2	79.5	-	Elevated AST (12.8)	Chen, S. (2020)	[63]

MVI: microvascular invasion; EHS: extra-hepatic spread; CRCLM: colorectal cancer liver metastases; DFS: disease-free survival; TTF: time to treatment failure; AST: alanine aminotransferase; ALT: alanine aminotransferase; CDDP: cisplatin; *: Severe adverse events.

## Abbreviations

The following abbreviations are used in this manuscript:

HCC	Hepatocellular carcinoma
TACE	Transarterial chemoembolization
HAIC	Hepatic artery infusion chemotherapy
GDA	Gastroduodenal artery
AEs	Adverse events
ECOG	Eastern Cooperative Oncology Group
ORR	Objective response rates
OS	Overall survival
PFS	Progression-free survival
PVTT	Portal vein tumor thrombosis
